# Administration of pilocarpine by microneedle patch as a novel method for cystic fibrosis sweat testing

**DOI:** 10.1002/btm2.10222

**Published:** 2021-04-03

**Authors:** Song Li, Kelsey Hart, Natalie Norton, Clare A. Ryan, Lokesh Guglani, Mark R. Prausnitz

**Affiliations:** ^1^ School of Chemical and Biomolecular Engineering Georgia Institute of Technology Atlanta Georgia USA; ^2^ Department of Large Animal Medicine University of Georgia College of Veterinary Medicine Athens Georgia USA; ^3^ Center for Cystic Fibrosis and Airways Disease Research Emory University Department of Pediatrics and Children's Healthcare of Atlanta Atlanta Georgia USA

**Keywords:** cystic fibrosis, drug delivery, iontophoresis, microneedle, pilocarpine, sweat test

## Abstract

The sweat test is the gold standard for the diagnosis of cystic fibrosis (CF). The test utilizes iontophoresis to administer pilocarpine to the skin to induce sweating for measurement of chloride concentration in sweat. However, the sweat test procedure needs to be conducted in an accredited lab with dedicated instrumentation, and it can lead to inadequate sweat samples being collected in newborn babies and young children due to variable sweat production with pilocarpine iontophoresis. We tested the feasibility of using microneedle (MN) patches as an alternative to iontophoresis to administer pilocarpine to induce sweating. Pilocarpine‐loaded MN patches were developed. Both MN patches and iontophoresis were applied on horses to induce sweating. The sweat was collected to compare the sweat volume and chloride concentration. The patches contained an array of 100 MNs measuring 600 μm long that were made of water‐soluble materials encapsulating pilocarpine nitrate. When manually pressed to the skin, the MN patches delivered >0.5 mg/cm^2^ pilocarpine, which was double that administered by iontophoresis. When administered to horses, MN patches generated the same volume of sweat when normalized to drug dose and more sweat when normalized to skin area compared to iontophoresis using a commercial device. Moreover, both MN patches and iontophoresis generated sweat with comparable chloride concentration. These results suggest that administration of pilocarpine by MN patches may provide a simpler and more‐accessible alternative to iontophoresis for performing a sweat test for the diagnosis of CF.

**Abbreviations**
CF: cystic fibrosis
HPLC: high‐performance liquid chromatography
MN: microneedle
PDMS: polydimethylsiloxane
PVA: poly(vinyl alcohol)

## 1 INTRODUCTION

Cystic fibrosis (CF) is the most common life‐limiting autosomal recessive genetic disorder and causes significant pulmonary and gastrointestinal problems.^1^ The genetic defect leads to diminished activity of an epithelial cell surface protein called Cystic Fibrosis Transmembrane Conductance Regulator that acts as an ion channel for the transport of chloride and bicarbonate.^2^ Early diagnosis of CF has been made possible by universal screening for CF based on elevated serum concentration of immunoreactive trypsinogen. All newborns with a positive newborn screen for CF have been referred to undergo sweat testing in the United States since 2010, and early detection and treatment of CF has improved the long‐term growth and respiratory outcomes of those affected.^3^

The sweat test procedure in newborn infants involves collection of sweat from both forearms after stimulation of the sweat glands with pilocarpine nitrate (a cholinergic agonist) delivered into the skin via iontophoresis.^4^ The delivery of pilocarpine into skin is intended to ensure a consistent rate of sweating and provide a small volume of sweat (generally 15–100 μl). In many instances, inadequate volumes of sweat are collected, necessitating repeat testing. This failure of adequate sweat collection is especially common when the sweat test is performed on infants less than 3 months of age, and may be related to several factors such as gestational age,^5^ ethnicity,^6^ and skin factors^7^ that can result in inadequate sweat collection. This can lead to delays in diagnosis and treatment, and also causes significant anxiety^8^ for the parents of the newborn who are waiting to find out if their child has CF.^9^

The current technique for sweat test has remained unchanged since it was first standardized in the 1960s.^10^ To improve the yield of sweat in newborn infants during the sweat test and to reduce diagnostic uncertainty, there is an urgent need to develop more accessible and simple‐to‐administer alternatives for inducing and collecting sweat. Such methodology will facilitate expedient and accurate diagnosis of CF in infants. The Cystic Fibrosis Foundation recommends that the rate of inadequate collection should be less than 10% of all sweat tests performed in children less than 3 months of age, and it should be less than 5% of all sweat tests done in children older than 3 months of age and adults. However, many clinical labs tend to struggle with meeting those targets, especially for the infants less than 3 months of age.

As an alternative to conventional iontophoresis, we propose pilocarpine administration by using a microneedle (MN) patch, which has been shown to deliver a variety of different drugs into the skin.^11^, ^12^, ^13^, ^14^ MN patches consist of an array of solid, conical needles measuring hundreds of microns in length that can be loaded with drugs. Upon application to skin, MNs penetrate across epidermis and into the dermis, where they dissolve and release their drug payload in the skin.^15^, ^16^ This procedure is minimally invasive, not painful and generally well tolerated.^17^, ^18^ In addition, application of MN patches is much easier than hypodermic injection or iontophoresis, and it can be performed after brief training.^19^, ^20^, ^21^ MN patches have been studied in many clinical trials for administration of zolmitriptan for treatment migraine,^22^ delivery of parathyroid hormone for treatment of osteoporosis,^23^ administering influenza vaccine^20^, ^24^ and delivery of other compounds,^13^ and are widely used for delivery of cosmetic products.^25^ Cost of manufacturing MN patches is expected to be less than US$1.00.^26^

Topical delivery of pilocarpine by MN patches has received limited prior attention. We previously conducted a study using metal MNs as a drug‐free pretreatment of the skin to create micropores that enhance the efficiency of pilocarpine delivery by iontophoresis.^27^ In another study, pilocarpine‐coated metal MNs were used to administer pilocarpine to the eye for cholinergic effects.^28^ However, the development and use of MNs to directly administer pilocarpine into the skin at levels suitable for sweat induction has not been described before.

The objectives of this study were to develop dissolvable MN patches for intradermal administration of pilocarpine and to determine their efficacy in inducing sweating using an animal (equine) model. The horse is one of the few animals, other than primates, that sweat and was therefore an appropriate animal model for this study (see Supporting Information for additional information).^29^, ^30^, ^31^ To the best of our knowledge, this work is the first study to develop dissolvable MN patches that load and deliver pilocarpine to skin for the purpose of sweat testing, and to assess the sweat induction of pilocarpine‐loaded MN patches in comparison with conventional iontophoresis.

## 2 RESULTS AND DISCUSSION

### 2.1 Characterization of MN patches and iontophoretic Pilogel disks

**FIGURE 1:**
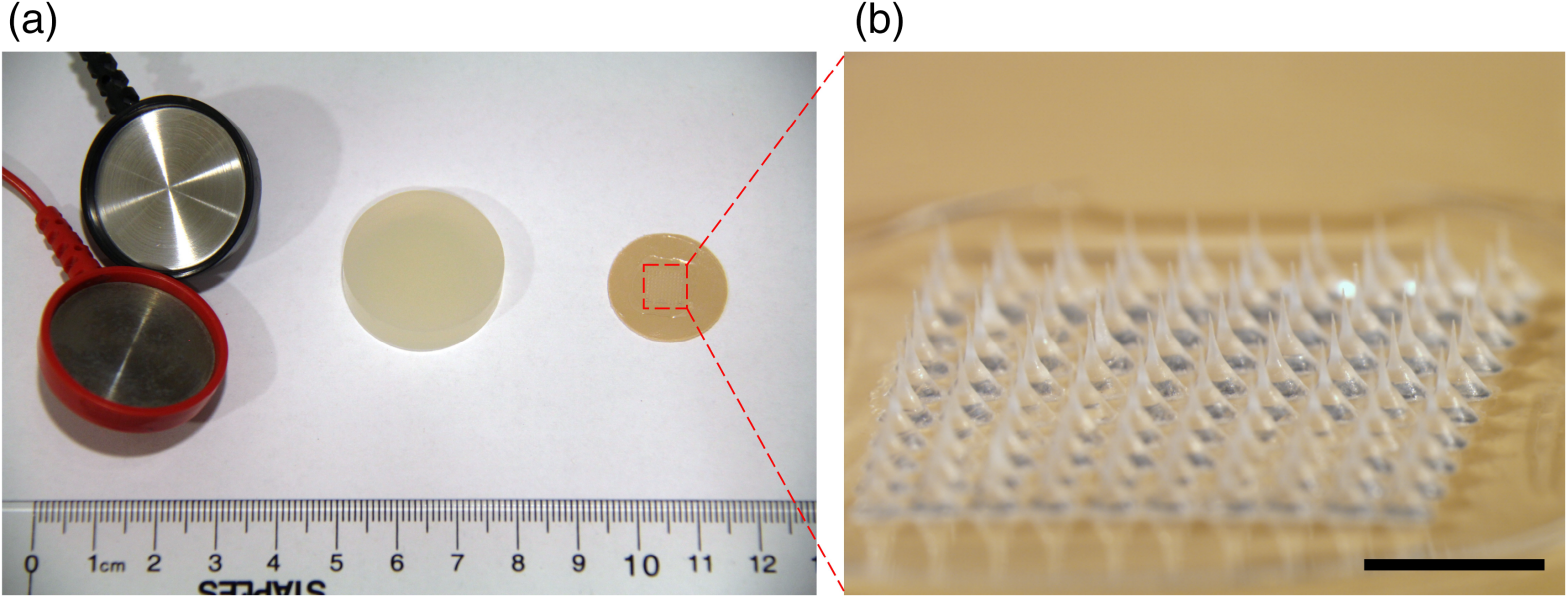
Comparison of pilocarpine delivery device used in iontophoresis and pilocarpine‐loaded microneedle patch. (a) Photograph of iontophoresis electrodes of a Webster Sweat Inducer (left), a Pilogel Iontophoretic Disk (middle), and a microneedle patch containing pilocarpine (right). The red frame indicated the appropriate area of microneedle array. (b) Microscopic view of a 10 × 10 array of microneedles in a patch. Scale bar is 2 mm

MN patches were designed to encapsulate pilocarpine within a water‐soluble matrix forming a conical shape with a sharp tip to facilitate penetration into skin (Figure 1). Each MN patch consisted of a 10 × 10 array of MNs arranged within a square with approximately 7 mm sides (i.e., ~0.5 cm^2^). In contrast, the iontophoretic Pilogel disks were circular with a diameter of 2.72 cm (i.e., ~5.8 cm^2^), thereby contacting an area of skin more than 10 times larger than the MN patch.

**FIGURE 2:**
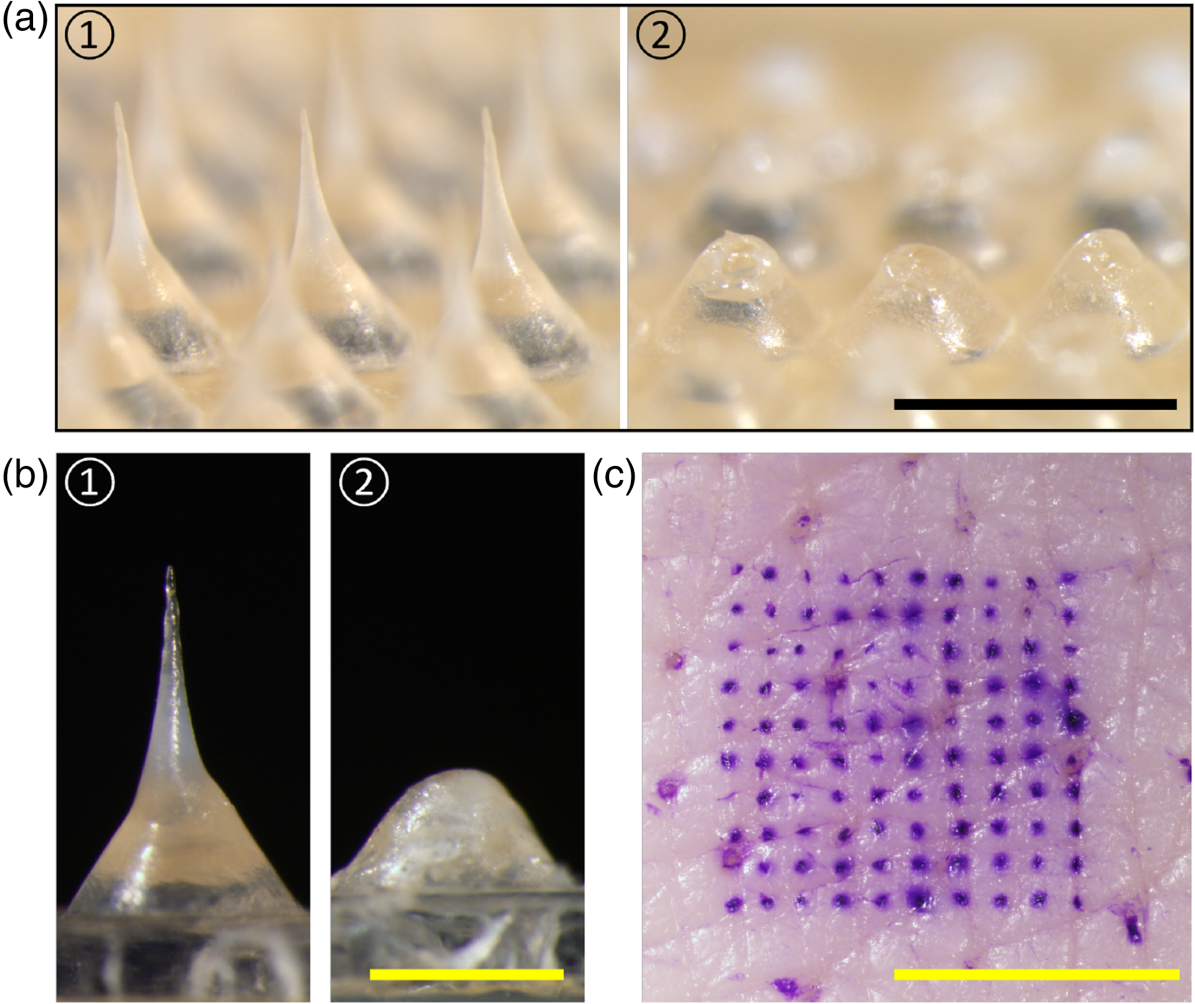
Microscopic imaging of pilocarpine‐loaded microneedle patches before and after application to the skin. Representative bright‐field microscopy images of (a) part of an array of microneedles and (b) an individual microneedle before (1) and after (2) application to porcine skin ex vivo, and (c) stained porcine skin ex vivo after application of microneedle patch, showing the micropores created by microneedle puncture. Scale bars are (a) 1 mm, (b) 0.5 mm, and (c) 5 mm

For microscopic examination as shown in Figure 2a,b, each conical MN (base diameter ≈ 200 μm, height ≈ 600 μm) was mounted atop an expanding pedestal (base diameter ≈ 600 μm, height ≈ 400 μm). The MNs were loaded with pilocarpine as well as poly(vinyl alcohol) (PVA) polymer to provide mechanical strength and sucrose to facilitate MN dissolution in the skin. After application to porcine skin ex vivo, the MNs dissolved in the skin, leaving only the base pedestals (Figure 2a,b), indicating that the pilocarpine loaded in the MNs was successfully delivered into the skin during MN patch application. Treating the skin with a dye that selectively stains sites of skin puncture revealed an array of MN‐generated micropores with the same 10 × 10 array geometry as the MN patch (Figure 2c), further indicating the ability of MNs to penetrate the skin.

In the current formulation, pilocarpine comprises 40% of the solids content in the MNs. The total amount of pilocarpine loaded per MN patch was measured as 500 ± 48 μg (*n* = 4). After application to porcine skin ex vivo, the residual pilocarpine content per MN patch was 237 ± 73 μg (*n* = 6), indicating that the delivered dose was ~263 μg (i.e., ~526 μg/cm^2^) and the delivery efficiency was ~53%. This incomplete delivery is likely due to the slow dissolution of PVA polymer, which comprised 40% of the solids content in the MNs. However, the high level of PVA used in the formulation is required to provide sufficient mechanical strength to the MNs to withstand the process of insertion into skin. Future studies could further optimize formulation to make MN dissolution faster and more complete, which would increase delivery efficiency.

The iontophoretic Pilogel disks were considerably larger and therefore had much higher pilocarpine loading amount, measured as 15.94 ± 0.27 mg (*n* = 3) (see Supporting Information for measurement). After iontophoresis on porcine skin for 10 min, the used disks contained 14.56 ± 0.15 mg (*n* = 3) residual pilocarpine, which indicates the delivered dose by iontophoresis was ~1.38 mg (i.e., ~238 μg/cm^2^) and the delivery efficiency was 8.7%. The delivery efficiency of iontophoresis was significantly lower than that of the MN patch.

When the MN patches were applied to the skin on horses in vivo, more pilocarpine, 407 ± 46 μg (*n* = 13), was dissolved from the MN patches compared with the ex vivo measurements in porcine skin. An explanation for this difference may be that the sweat induced by the MN patch in the horse (but not in the porcine skin ex vivo) might have further dissolved the MNs at the skin surface, giving the appearance of greater pilocarpine delivery efficiency. The same effect might have also occurred at the iontophoresis sites. However, it is unclear whether the extra dissolved pilocarpine entered the skin and played a role in sweat induction. As a conservative comparison, we based our analysis of the results on the ex vivo data on the delivered pilocarpine dose from both MN patches and iontophoresis as 0.26 mg and 1.38 mg, respectively (Table 1).

**TABLE 1:** Comparison of parameters between microneedle patches and iontophoresis

	Microneedle patches	Pilocarpine iontophoresis disks
Application area (cm^2^)	0.5^a^	5.8^b^
Pilocarpine dose (mg)^c^	0.26 ± 0.07	1.38 ± 0.15
Pilocarpine/application area (mg/cm^2^)	0.52 ± 0.14	0.24 ± 0.03

^a^ Each microneedle patch was square in shape with side length of ~0.7 cm.

^b^ Each pilocarpine iontophoresis disk was round in shape with a diameter of ~2.72 cm.

^c^ The dose was calculated as the difference between unused and used devices (i.e., microneedle patches or pilocarpine disks).

### 2.2 Comparison of sweat volume induced by MN patches and iontophoresis

**FIGURE 3:**
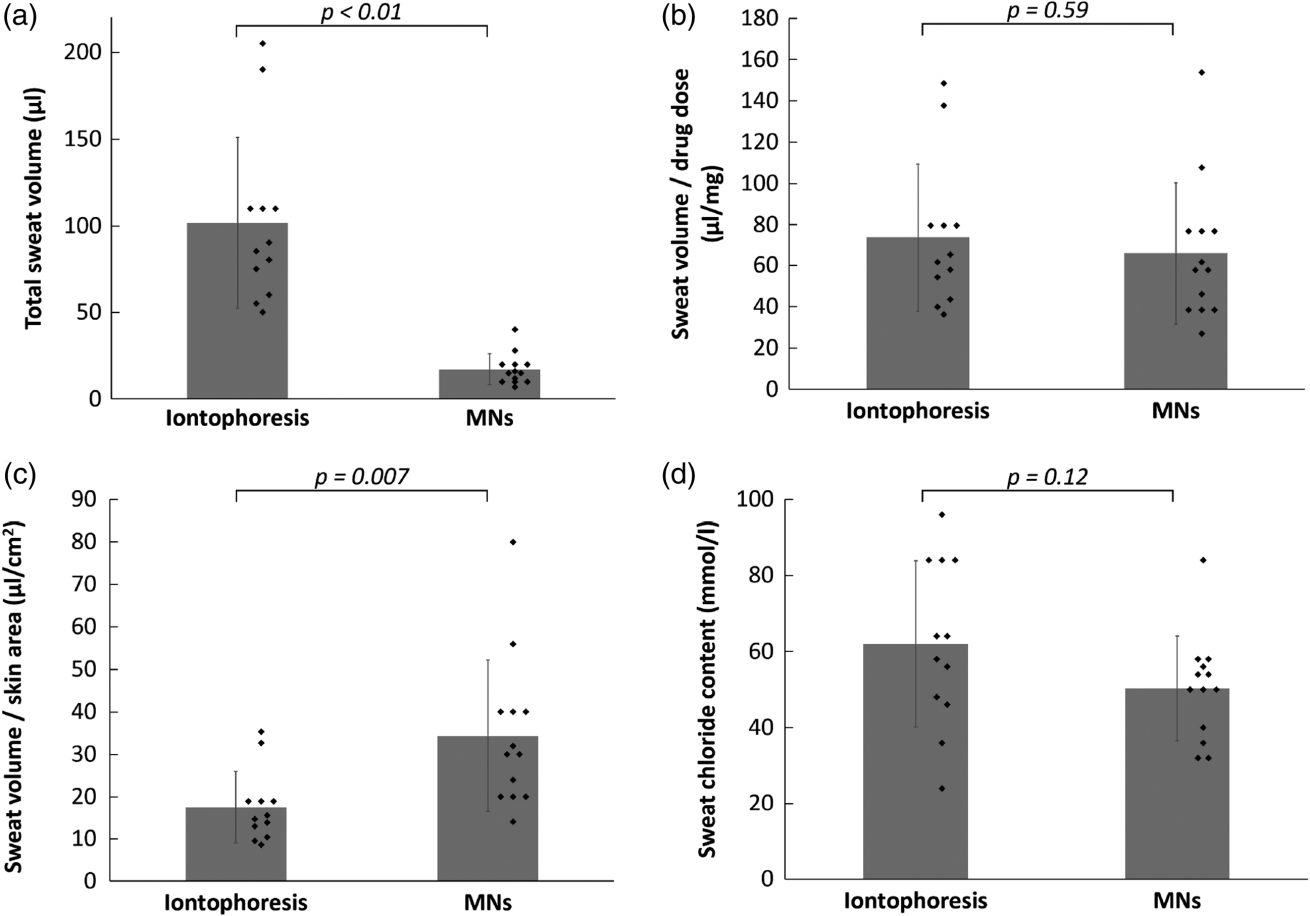
Comparison of sweat volumes and sweat chloride contents induced by pilocarpine delivery by microneedle (MN) patches (*n* = 13) and iontophoresis (*n* = 12) in horses. (a) Total volume of sweat collected. (b) Sweat volume collected per unit of pilocarpine dose. (c) Sweat volume collected per unit of skin area. (d) Chloride content of collected sweat. Data represent mean ± SD

Sweat samples were collected from 25 sites on the neck of four horses after introducing pilocarpine by either MN patches or iontophoresis. No changes to physical exam parameters, cervical skin irritation, or other adverse effects were noted in any horses during or after the study. From all application sites, at least 10 μl of sweat was collected. The average total sweat volume from an iontophoresis site was 101 ± 49 μl over a pilocarpine application area of 5.8 cm^2^, corresponding to a sweat collection density of 17 ± 8 μl/cm^2^. The average total sweat collected from an MN patch site was 17 ± 8 μl over a pilocarpine application area of 0.5 cm^2^, corresponding to a sweat collection density of 34 ± 16 μl/cm^2^ (Figure 3a,c). We believe that sweat density is the most appropriate basis for comparison between the two techniques because sweat production is expected to scale directly with area and because sweat collection is usually done over a standard area of skin using a sweat collection device like the Macroduct Sweat Collector used here.

While the total amount of sweat collected from the iontophoresis sites was greater than that collected from the MN patch sites (Figure 3a), when accounting for the different pilocarpine application areas, the sweat collection density from the MN patch sites was 2.0‐fold greater than that collected from the iontophoresis sites (Figure 3c). This ratio was relatively consistent on each of the four horses (2.3‐, 1.6‐, 2.1‐, and 1.6‐fold greater). This suggests that the difference between MN patches and iontophoresis on sweat induction was not determined by the individual differences between horses. Instead, the difference in sweat collection density appears to mainly reflect the different sweat‐inducing abilities of the two pilocarpine delivery procedures.

To help explain why the sweat collection densities differed between the two methods, we calculated the sweat volume per unit of pilocarpine dose delivered to the skin. This analysis revealed no significant difference between iontophoresis (73 ± 36 μl/mg) and MN patches (66 ± 34 μl/mg) (Figure 3b). However, because the amount of pilocarpine delivered per unit area of skin was 2.2‐fold greater when administered by MN patches (~526 μg/cm^2^) compared to iontophoresis (~238 μg/cm^2^), this likely accounts for the greater sweat collection density seen after pilocarpine delivery by MN patch. This further indicates that using MN patches to deliver pilocarpine has a comparable or possibly better sweat‐inducing capability as the traditional iontophoresis.

It should be noted that although sweat collection density was greater using a MN patch, the MN patch induced less total sweat volume than iontophoresis. Because the MN patch delivered twice as much pilocarpine per unit area, we expect that a larger MN patch with the same area as the pilocarpine disk used for iontophoresis (i.e., 5.8 cm^2^) would correspondingly deliver twice as much pilocarpine and thereby induce more total sweat volume compared to iontophoresis, because sweat production is known to increase with pilocarpine dose delivered.^32^, ^33^ Additional studies will be needed to develop larger MN patches and measure the resulting sweat production.

### 2.3 Comparison of sweat chloride content induced by MN patches and iontophoresis

Chloride contents in the iontophoresis‐induced sweat (60.0 ± 21.8 mmol/L) and MN patch‐induced sweat (50.3 ± 13.8 mmol/L) were not significantly different (Figure 3d), indicating that the method of pilocarpine administration (iontophoresis vs. MN patch) did not significantly affect the chloride content in the collected sweat.

### 2.4 Significance to future clinical applications

This study demonstrates that MN patches are able to deliver pilocarpine to the skin of horses to induce sweating. The amount of sweat produced per dose of pilocarpine delivered, and the chloride concentration of that sweat, was similar for delivery of pilocarpine via MN patch and iontophoresis. This suggests that MN patch delivery could be a suitable alternative to iontophoresis delivery of pilocarpine. The pilocarpine dose delivered per unit area doubled with MN patch delivery compared to iontophoresis delivery. This suggests that use of a larger MN patch could produce larger amounts of sweat and/or potentially produce adequate amounts of sweat in less time compared to current iontophoretic methods.

This simple and low‐cost method of pilocarpine delivery by MN patch compared to iontophoresis could make sweat testing more widely available and no longer limited to use by specialty clinics. In addition, the larger pilocarpine dose per unit area could enable MN patch delivery to more consistently generate the amount of sweat required to perform a chloride measurement, thus potentially making the sweat test more reliable and avoiding the need for repeated measurement attempts.

The fact that MN patches are generally perceived as painless^20^, ^34^, ^35^ may also make this method of pilocarpine delivery more attractive to healthcare providers, patients, and their caregivers. Numerous studies have shown that adults, children, parents, and clinicians prefer MN patch delivery over hypodermic injection.^35^, ^36^, ^37^, ^38^ When human subjects experienced both MN patch application to the skin and iontophoretic sweat testing in a prior study, there was no significant difference in reported pain between the two procedures.^27^ Placebo MN patches, as well as MN patches administering various drugs and vaccines, have been shown to be well tolerated, with only transient, mild erythema reported as the most common side effect.^17^, ^18^, ^20^, ^21^, ^35^ Previous studies have also shown that MN patches are so simple to apply that naïve human subjects can successfully apply them after only brief training.^19^, ^20^, ^21^

## 3 MATERIALS AND METHODS

### 3.1 Fabrication of MN patches

The pilocarpine‐loaded MN patches were fabricated by a two‐step molding process using polydimethylsiloxane (PDMS) molds based on an established method.^39^ The first casting solution was a mixture of 10% (wt/vol) pilocarpine nitrate (Sigma Aldrich), 10% (wt/vol) PVA (4‐88, Millipore Sigma) and 5% (wt/vol) sucrose (Sigma Aldrich), which was prepared in deionized water. This solution was cast on PDMS molds under vacuum to facilitate filling the solution into the mold cavities to form the MNs. After 20 min, excess solution was removed, and the filled molds were centrifuged at 5000*g* for 20 min to dry the drug‐loaded MNs. The second casting solution containing 20% (wt/wt) polystyrene in 1,4‐dioxane was then cast on the filled PDMS molds under vacuum to form the patch backing. The molds were kept under vacuum for another 3 h to dry the solution at room temperature, and then further dried at 40°C overnight before demolding the MN patches using adhesive tapes. Each patch was immediately sealed in individual pouches with desiccant and stored at room temperature until use.

### 3.2 Characterization of MN patches in vitro

MN patches were applied to shaved porcine skin ex vivo to study their skin insertion properties before application on horses in vivo. Briefly, a MN patch was manually pressed against the porcine skin by thumb for ~10 s, and then left in place for 20 min to allow MN dissolution and release of drugs in the skin. After being removed from the skin, patches were saved for further examination. To visualize the sites of MN penetration into skin, a Gentian violet solution (Humco) was applied to the MN patch application site and left there for 5 min prior to cleaning with isopropyl alcohol wipes. This permitted selective staining of the micropores created by the MNs. The stained skin as well as the morphology of used MN patches was examined under a microscope (Olympus SZX16, Tokyo, Japan) to assess the efficiency of MN penetration into skin and the morphology of MNs after dissolution in the skin.

The pilocarpine dose delivered by MN patches was calculated as the difference between the pilocarpine contents in patches before and after application to skin. The pilocarpine content in MN patches was measured by high‐performance liquid chromatography (HPLC) (Agilent 1200) after dissolving the patch in a known volume of deionized water. The HPLC assay used 10% methanol and 90% water containing 0.1% trifluoroacetic acid as the mobile phase, and an Eclipse XDB‐C18 (5 μm, 4.6 × 150 mm; Agilent) column as the solid phase. The peak area under the curve was used to determine pilocarpine concentration (calibrated based on pilocarpine solutions of known concentration, 50–1000 μg/ml) and thereby determine pilocarpine content in MN patches.

### 3.3 Animals and preparation for in vivo studies

Eight healthy outbred adult horses were used for the in vivo portions of this study. Specifically, animals included six castrated males and two intact females of various breeds, with a median age of 15 years (range 4–23 years) and weight ranging 488–594 kg. The horses were from University of Georgia College of Veterinary Medicine equine research herd and had no history of illness within the 2 months prior to the study or any known sweating disturbance. Systemic health was confirmed prior to and during the study by serial physical examination. Twenty‐four hours prior to sampling, horses were brought into University of Georgia Veterinary Teaching Hospital and housed in a climate‐controlled barn (ambient temperature 20–25°C) for acclimatization and remained in this facility for the entire testing period. Animals were housed individually in stalls and had ad libitum access to grass hay and water during the sampling period. During sample collection, horses were restrained without sedation in stocks and allowed to eat grass hay as desired to ensure compliance. All horses used in this study were accustomed to handling and restraint and remained calm during sampling; sedation was specifically avoided due to potential neuroendocrine effects that could impact sweat responses.

Immediately prior to all testing, the entire cervical region was clipped bilaterally with electric clippers (#40 blades). On the right cervical region, three 3 × 3 cm areas were further shaved manually with a straight razor to permit good contact of the MN patches, iontophoretic pilocarpine disks and sweat collection pads to the skin. The skin was then washed with a mild detergent soap and dried thoroughly with a cotton towel to remove skin oils and facilitate uniform adherence of sweat induction and collection devices.

Horses' vital parameters (temperature, pulse, respiratory rate), behavior, and cervical skin were monitored for any adverse effects before testing, every 10 min during the pilocarpine administration and sweat collection period and then 6, 12, and 24 h after sample collection was completed.

### 3.4 Application of MN patches and iontophoresis to induce sweating in vivo

As a preliminary experiment, we first confirmed pilocarpine‐induced sweat production in horses and optimized small volume sweat collection in two horses (see Supporting Information). Pilocarpine‐induced sweat production via MN patches and iontophoresis was then compared in four horses after acclimatization and preparation as above. In each animal, three MN patches were applied manually by thumb pressure to the right side of the neck, approximately 5 cm apart, and left in place for 20 min. Concurrently, on the left side of the neck, Pilogel Iontophoretic Disks (Ref SS‐032, ELITechGroup) were mounted onto electrodes, and pilocarpine was delivered via iontophoresis (Webster Sweat Inducer, model 3700‐sys, ELITechGroup) for 10 min (two machine cycles) in two separate locations sequentially following the manufacturer's instructions.

After completion of iontophoresis and removal of the MN patches, sweat was collected to quantify volume and chloride concentration. In pilot studies, the conventional Macroduct Sweat Collector (ELITechGroup) used for sweat collection in people could not be consistently adhered to the convex cervical region on the horse, leading to unreliable and inconsistent sweat collection. Thus, a modified sweat collection protocol was developed using single layer cotton gauze pads (Dukal) covered by a similarly sized piece of 150 μm‐thick polypropylene plastic sheeting and secured under a piece of heavy‐duty adhesive tape (approximately 5 × 15 cm, Black Gorilla Tape, Gorilla Glue Co.).

Gauze pads to collect MN‐induced and iontophoresis‐induced sweat were 1 cm^2^ and 2 cm^2^, respectively. Plastic sheeting approximately 1 mm larger in length and width was applied over the gauze pads. After 30 min, the gauze pads were collected and immediately weighed on a microbalance to calculate sweat volume by subtracting the dry weight. Gauze pads were then immediately placed inside a 3 ml polypropylene syringe barrel inserted in a 15 ml conical polypropylene tube (Becton, Dickinson and Company) and sealed prior to centrifuging at 1100*g* for 10 min. Sweat recovered after centrifugation was collected into a polyproylene microcentrifuge tube (USA Scientifics) and frozen at −80°C until analysis for chloride concentration.

### 3.5 Sweat chloride quantification via chloridometer

All sweat samples were batch‐analyzed at the end of the study period after thawing to room temperature. Because horse sweat chloride concentration is much higher than that of human sweat, all sweat samples from horses were diluted 4‐fold with distilled water prior to measurement of chloride concentration. Chloride concentration of the collected sweat samples was measured using a chloridometer (ChloroChek®, Wescor). The measurement requires a minimum volume of 10 μl of sweat and is based on the principle of coulometric titration. Following the manufacturer's instructions, the sweat sample was added to an acid buffer/working solution into which silver electrodes were immersed. The results obtained for the horse sweat samples were then converted to final values by multiplying by 4 to account for the dilution performed prior to testing.

### 3.6 Statistics

All data are presented as mean ± SD. Total sweat volume, sweat volume/drug dose, sweat volume/skin area, and sweat chloride concentration were compared between MN and iontophoresis sites with two‐tailed unpaired Student's *t* tests. Statistical significance was set at *p* ≤ 0.05 for all comparisons.

## 4 CONCLUSIONS

This study investigated the feasibility of using MN patches to deliver pilocarpine and induce sweat for chloride quantification, to test the expectation that MN patch delivery can make sweat testing simpler and more widely available than current iontophoresis‐based methods. By comparing pilocarpine delivery by MN patches and iontophoresis on horses, we found that MN patches could consistently cause sweat production, and that the sweat collection density generated using MN patches was approximately double that generated using conventional iontophoresis. This greater sweat density could be explained by the amount of pilocarpine delivered per unit area of skin, which was approximately twice as large after MN patch administration compared to iontophoresis. Because skin contact area of pilocarpine iontophoretic disks was much larger than MN patches, the total amount of sweat produced after iontophoretic delivery was greater than for MN patches. However, the data suggest that if a MN patch of similar skin contact area were used, it would likely generate comparable or more sweat than conventional iontophoresis. Moreover, both sweat‐inducing methods generated sweat of similar chloride content and did not cause any adverse local or systemic effects. These results suggest that pilocarpine‐containing MN patches could offer a simple and more accessible alternative for sweat induction to support efficient and minimally invasive CF diagnosis in infants and children. The future optimization of MN patches for this use may be focused on increasing MN patch application area, and confirmation of these experimental findings in human clinical trials.

## CONFLICT OF INTEREST

M. R. P. is an inventor of patents licensed to companies developing microneedle‐based products, is a paid advisor to companies developing microneedle‐based products, and he is a founder/shareholder of companies developing microneedle‐based products (Micron Biomedical). This potential conflict of interest has been disclosed and is managed by Georgia Tech.

## AUTHOR CONTRIBUTIONS

**Song Li:** Data curation; formal analysis; investigation; methodology; project administration; writing‐original draft; writing‐review & editing. **Kelsey Hart:** Investigation; methodology; project administration; supervision; writing‐review & editing. **Natalie Norton:** Investigation; methodology; project administration; writing‐review & editing. **Clare Ryan:** Investigation; methodology; writing‐review & editing. **Lokesh Guglani:** Conceptualization; data curation; formal analysis; funding acquisition; investigation; methodology; project administration; resources; supervision; validation; writing‐original draft; writing‐review & editing. **Mark Prausnitz:** Conceptualization; data curation; formal analysis; funding acquisition; investigation; methodology; project administration; resources; supervision; writing‐review & editing.

## DISCLOSURE

The fabrication and use of microneedles made out of pilocarpine for the induction of sweat production during sweat testing has been submitted as a patent to the United States Patent and Trademark Office (Patent Pending).

## Supporting information

**Appendix S1** Supporting Information.

## Data Availability

The animal data included in this article has not been uploaded to any public databases or archives. There is no human subject data in this article.
