# Supplementary material for: Administration of pilocarpine by microneedle patch as a novel method for cystic fibrosis sweat testing
Source: Bioeng Transl Med. 2021 Apr 3;6(3):e10222. doi: 10.1002/btm2.10222 (PMC8459588; doi:10.1002/btm2.10222)
Supplement: Supplementary file 1 — Appendix S1 Supporting Information. [file BTM2-6-e10222-s001.docx]

**Supplementary Information**

**Administration of pilocarpine by microneedle patch as a novel method for cystic fibrosis sweat testing**

Song Li,^1^ Kelsey Hart,^2*^ Natalie Norton,^2^ Clare A. Ryan,^2^ Lokesh Guglani,^3*^ Mark R. Prausnitz^1*^

^1^ School of Chemical & Biomolecular Engineering, Georgia Institute of Technology, Atlanta, GA 30332 USA;

^2^ Department of Large Animal Medicine, University of Georgia College of Veterinary Medicine, Athens, GA 30602 USA;

^3^ Center for Cystic Fibrosis and Airways Disease Research, Emory University Department of Pediatrics and Children’s Healthcare of Atlanta, Atlanta, GA 30322 USA.

*Corresponding authors and their addresses for correspondence:

Kelsey Hart DVM PhD DACVIM (LAIM)

Email: [khart4@uga.edu](mailto:khart4@uga.edu)

Associate Professor, Large Animal Internal Medicine

Director, DVM-PhD Program

2200 College Station Road

Veterinary Medical Center

University of Georgia College of Veterinary Medicine

Athens, GA 30602. Phone: 706-542-3223; Fax: 706-357-0108

Lokesh Guglani MD FAAP

Email: [lokesh.guglani@emory.edu](mailto:lokesh.guglani@emory.edu)

Associate Professor of Pediatrics

Division of Pulmonology, Allergy/Immunology, Cystic Fibrosis, and Sleep

Department of Pediatrics, Emory University School of Medicine

Children’s Healthcare of Atlanta - Egleston

2015 Uppergate Drive, Atlanta, GA 30322. Phone: 404-712-2324, Fax: 404-712-0920

Mark R. Prausnitz PhD

Email: [prausnitz@gatech.edu](mailto:prausnitz@gatech.edu)

Regents' Professor and J. Erskine Love Jr. Chair in Chemical & Biomolecular Engineering

School of Chemical and Biomolecular Engineering

Georgia Institute of Technology

311 Ferst Drive

Atlanta, GA 30332-0100 USA. Phone: +1 (404) 894-5135; Fax: +1 (404) 894-2291.

1. Preliminary studies of sweat induction by pilocarpine in horses

Because sweat induction via pilocarpine is not well described in horses, a preliminary pilocarpine dose-response study was conducted to confirm that proposed doses to be administered via MN patch and iontophoresis would adequately induce sweating in horses. After acclimation as explained in the Methods section of the manuscript, 0.1 ml of serial dilutions of pilocarpine were injected intradermally in the cervical region of 2 horses, and sweat production was quantified as described in the Methods section of the manuscript. Pilocarpine for intradermal injection was prepared from the same pilocarpine powder used to load the MN patches, and was reconstituted and diluted with sterile water at 1, 2, 5, 10, and 20 mg/ml. Sterile water was injected as a negative control. Sweat was collected with gauze pads as described in the Methods section of the manuscript.

Next, because sweat production induced via pilocarpine delivery by iontophoresis is not described in horses, this procedure was optimized for equine use in a preliminary study on 2 horses. Both positive and negative electrodes of the Macroduct device were loaded with Pilogel Iontophoretic Discs (Ref SS-032, ELITechGroup, Logan, UT), and were adhered to the cervical region using heavy-duty adhesive tape (Gorilla tape, Gorilla Glue Co., Cincinnati, OH). The standard 5-minute cycles of the iontophoresis device (Webster Sweat Inducer, model 3700-sys, ELITechGroup) were initiated and the skin under the positive electrode was monitored for visible sweat production at the completion of each cycle. Two machine cycles (10 minutes total time) were required for visible sweat production at the electrode site, after which Macroduct collectors for sweat collection were secured in place as described in the Methods section of the manuscript.

1. Horses as an animal model for sweating

Animal models for sweat test research are limited, as most laboratory and domestic animal species lack sweat glands entirely or have them limited to their digital pads (e.g., dogs and cats) ^1, 2^. Pigs have low numbers of sweat glands diffusely distributed throughout their epidermis, but the paucity of these glands limit this species’ utility for sweat research.^1^ In contrast, horses have substantial apocrine and eccrine sweat glands generally distributed throughout their skin surface and, like humans, use sweating as a primary method of thermoregulation.^1, 3^ Further, as in people, neuroendocrine control of equine sweating includes both adrenergic and cholinergic mechanisms ^3, 4^. With the exception of some non-human primate species (rhesus and patas monkeys) ^5^, horses represent the most suitable animal model for sweat studies.

1. Measurement of the pilocarpine dose delivered from Pilogel Iontophoretic Discs

After iontophoresis, the pilocarpine discs were cut into small pieces (around 3 mm in size), kept in centrifuge tubes and frozen at -20 °C overnight. The cut disc pieces were thawed at room temperature (20 – 25 °C) on the second day and 20 ml deionized water was added. Mixtures were sonicated in sonication bath for 2 h. The pieces were incubated in the solution and stirred at 300 rpm for another day. The supernatant was collected by centrifugation at 3000 g for 5 min for HPLC analysis of pilocarpine content, as described in the Methods section of the manuscript The dose of pilocarpine administered by iontophoresis was calculated as the difference between pilocarpine content of discs before and after iontophoresis.

**References**

1. Collier RJ, Gebremedhin KG. Thermal Biology of Domestic Animals. *Annual Review of Animal Biosciences*. 2015/02/16 2015;3(1):513-532. doi:10.1146/annurev-animal-022114-110659

2. Best A, Kamilar JM. The evolution of eccrine sweat glands in human and nonhuman primates. *Journal of Human Evolution*. 2018/04/01/ 2018;117:33-43. doi:<https://doi.org/10.1016/j.jhevol.2017.12.003>

3. Hodgson DR, Davis RE, McConaghy FF. Thermoregulation in the horse in response to exercise. *British Veterinary Journal*. 1994/05/01/ 1994;150(3):219-235. doi:<https://doi.org/10.1016/S0007-1935(05)80003-X>

4. Jenkinson DM, Elder HY, Bovell DL. Equine sweating and anhidrosis Part 1 – equine sweating. *Veterinary Dermatology*. 2006/12/01 2006;17(6):361-392. doi:10.1111/j.1365-3164.2006.00545.x

5. Elizondo RS. Primate models to study eccrine sweating. *American Journal of Primatology*. 1988/01/01 1988;14(3):265-276. doi:10.1002/ajp.1350140307
